# Renal Effects of Sulfated Polysaccharides from the Seaweed *Gracilaria cornea*

**DOI:** 10.3390/toxins17100499

**Published:** 2025-10-09

**Authors:** Terentia Batista Sá Norões, Sophia Moinhos, Helena Serra Azul Monteiro, Alice Maria Costa Martins, Ricardo Parente Garcia Vieira, Claudio Gleidiston Silva

**Affiliations:** 1 Department of Medicine, Centro Universitário Doutor Leão Sampaio (UNILEÃO), Maria Letícia Leite Pereira Ave., Lagoa Seca—Cidade Universitária, Juazeiro do Norte 63040-405, CE, Brazil; sophiafmoinhos@gmail.com; 2 Department of Physiology and Pharmacology, School of Medicine, Federal University of Ceara, Coronel Nunes de Melo St., 1127, Fortaleza 60430-275, CE, Brazil; hsazul@gmail.com (H.S.A.M.); martinsalice@gmail.com (A.M.C.M.); 3 Department of Medicine, Federal University of Cariri (UFCA), Ten. Raimundo Rocha Ave., 1639, Juazeiro do Norte 63048-080, CE, Brazil; ricardopgv@gmail.com (R.P.G.V.); claudiogleidiston@hotmail.com (C.G.S.)

**Keywords:** *Gracilaria cornea*, sulfated polysaccharides, isolated kidney perfusion, MDCK cells, apoptosis

## Abstract

Sulfated polysaccharides (SPs) are abundant in seaweed and have several industrial and biomedical applications, but their renal effects remain unclear. This study evaluated the effects of total sulfated polysaccharides (TSPs) from *Gracilaria cornea* using an isolated rat kidney perfusion model. TSP at 3 µg/mL increased perfusion pressure and renal vascular resistance at 90–120 min, while 4.5 µg/mL induced earlier and more pronounced changes (from 60 min). Urinary flow decreased at 1 µg/mL (90 min) but increased at 4.5 µg/mL (90–120 min). Sodium transport was reduced at all concentrations, whereas potassium and chloride transport remained unchanged. Histological analysis revealed protein deposits in tubules and urinary space, indicating tubular injury. In vitro, TSP reduced MDCK cell viability in a concentration-dependent manner and induced apoptosis, with some cells progressing to secondary necrosis. In conclusion, TSP altered renal physiology and morphology and triggered apoptotic pathways in renal cells, highlighting the need for further mechanistic and translational studies.

## 1. Introduction

Seaweeds inhabit a wide range of environments, including marine ecosystems, fresh-water bodies, soil surfaces, and even tree trunks. Beyond the pigments that define their coloration, these organisms are notable for their biochemical constituents of scientific and commercial value, such as hemagglutinins, which position them as promising sources of natural compounds for pharmacological exploration [1,2]. Rich in carbohydrates, seaweeds also serve industrial purposes as thickeners, emulsifiers, and stabilizers in numerous food formulations [3,4].

Among the red algae, *Gracilaria* spp. (family Gracilariaceae) is commonly found along tropical Atlantic coastlines and is a primary source of agar a polysaccharide-rich extract derived from agarophyte species [3,5]. In these macroalgae, sulfated carbohydrates, including sulfated galactans and fucans, are abundantly present and have garnered attention for their diverse biotechnological applications [4]. Sulfated polysaccharides (SPS) from marine algae have shown considerable potential in the pharmaceutical, cosmetic, and biotechnology industries due to their unique chemical and biological profiles [6].

Previous studies have reported a wide spectrum of bioactivities associated with SPs from marine algae, including antitumor [7,8,9], anticoagulant [10,11], antiviral [12,13], immunomodulatory [14], antithrombotic [15], anti-inflammatory, and antinociceptive effects [16]. These findings underscore their relevance not only for industrial applications but also as candidates for drug development.

However, despite the growing recognition of sulfated polysaccharides (SPS) from Gracilaria cornea as promising bioactive agents, their effects on renal physiology remain largely unexplored, leaving a significant knowledge gap. Furthermore, the characterization of renal responses to natural products is essential for the development of safe and effective therapeutic agents. Standardized methodologies for evaluating renal function in experimental models, such as measurements of serum creatinine, urea, urine output, and histopathological assessments, provide a robust framework for such investigations. Considering the widespread use of Gracilaria-derived products in the food industry in Brazil, particularly along the Ceará coastline, it is of great importance to assess whether bioactive compounds from this species might exert unforeseen renal effects. Therefore, this study was designed to test the hypothesis that total sulfated polysaccharides (TSPs) from *Gracilaria cornea* alter renal hemodynamics, electrolyte transport, and morphology in an ex vivo perfused kidney model, and induce cytotoxic/apoptotic effects in renal epithelial cells in vitro.

Considering the widespread use of Gracilaria-derived products in the food industry in Brazil, particularly along the Ceará coastline, it is of great importance to assess whether bioactive compounds from this species might exert unforeseen renal effects. This study aims to bridge this knowledge gap by investigating the renal actions of sulfated polysaccharides extracted from *Gracilaria cornea*, employing an integrated experimental approach.

## 2. Results

To evaluate the renal effects of TSP without systemic interference, an isolated rat kidney perfusion model was employed. The chemical composition of TSP is primarily composed of 3,6-anhydro-α-L-galactose, with secondary components including 6-O-methylgalactose, glucose, xylose, and sulfate residues [17].

**Figure 1:**
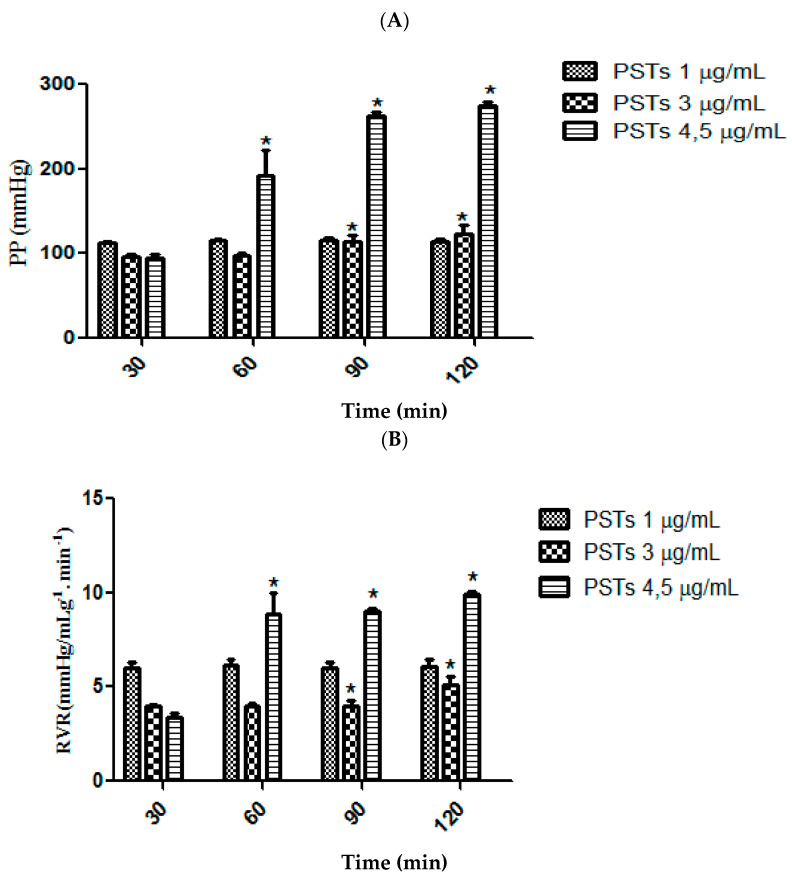

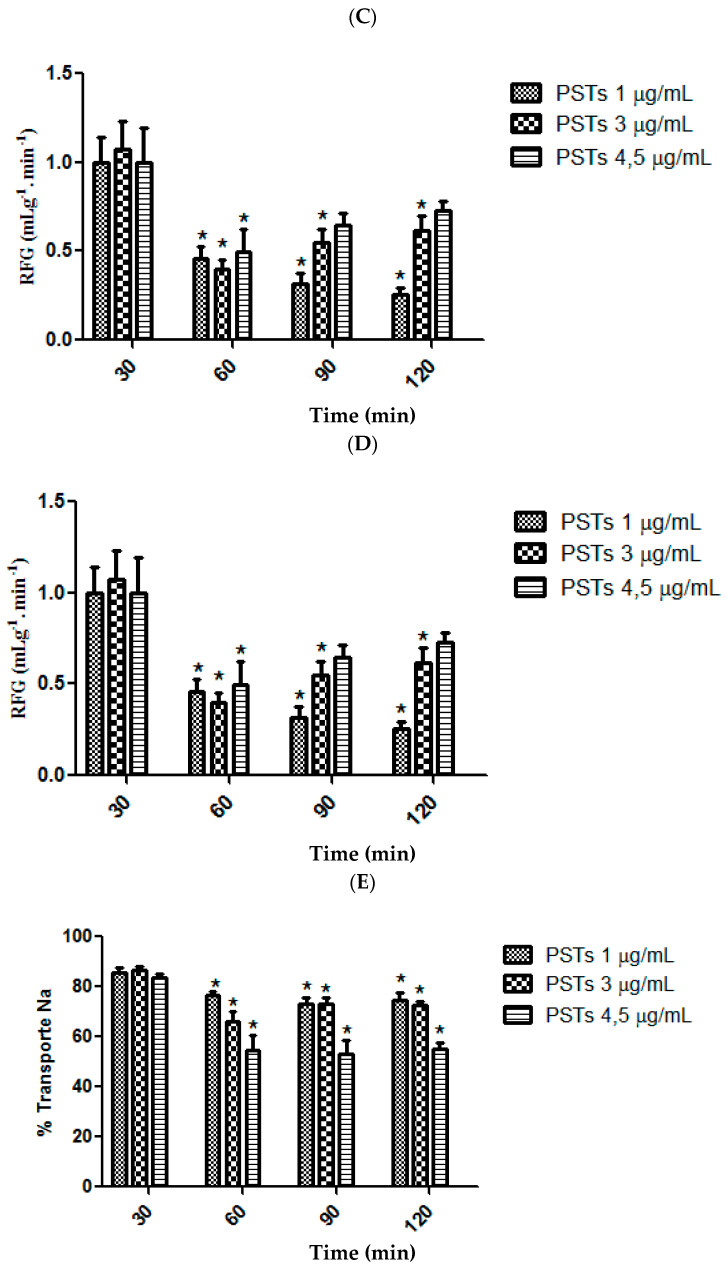

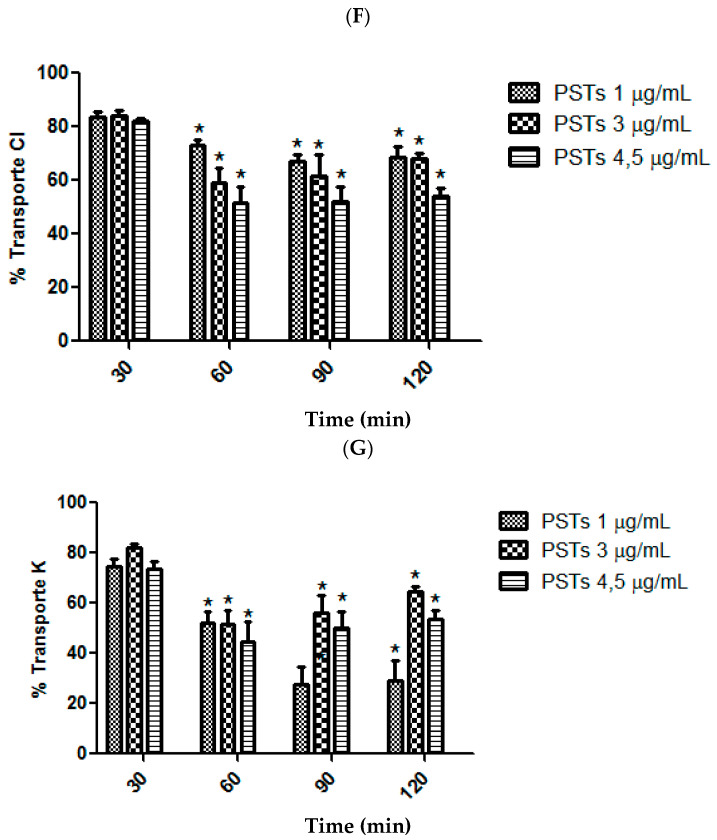
Effects of total sulfated polysaccharides (TSPs) from *Gracilaria cornea* (1.0, 3.0, and 4.5 μg/mL) on perfusion pressure (PP; (**A**)), renal vascular resistance (RVR; (**B**)), urinary flow (UF; (**C**)), glomerular filtration rate (GFR; (**D**)), and tubular transport of sodium (%TNa^+^; (**E**)), potassium (%TK^+^; (**F**)), and chloride (%TCl^−^; (**G**)). Data are expressed as mean ± S.E.M. (*n* = 6). Comparisons were made between time points (60, 90, and 120 min) and baseline (30 min) using One-Way ANOVA followed by Student’s *t*-test. Significance levels are indicated as *p* < 0.05, * *p* < 0.01.

At the lowest concentration (1 μg/mL), TSP did not significantly alter renal vascular parameters. However, at 3 μg/mL, there was a noticeable increase in perfusion pressure and renal vascular resistance at 90 and 120 min of perfusion (Figure 1A,B). The highest tested concentration (4.5 μg/mL) induced an earlier and more pronounced elevation in both parameters, observable from 60 min onward.

Urinary flow (UF) and glomerular filtration rate (GFR) responded differently. Even at the lowest concentration (1 μg/mL), TSP significantly reduced UF at 90 min. At 4.5 μg/mL, UF increased at 90 and 120 min, reaching 0.290 ± 0.017 and 0.331 ± 0.02 mL·g^−1^·min^−1^, equivalent to 52% and 73% of the internal control (C30 = 0.191 ± 0.03 mL·g^−1^·min^−1^), respectively. GFR was less affected, but exhibited a slight reduction at 60 min (Figure 1C,D).

TSP impaired tubular function, as shown by a decrease in sodium reabsorption (%TNa^+^) at all concentrations tested (Figure 1E). No significant changes were detected in chloride (%TCl^−^) or potassium (%TK^+^) transport (Figure 1F,G).

**Figure 2:**
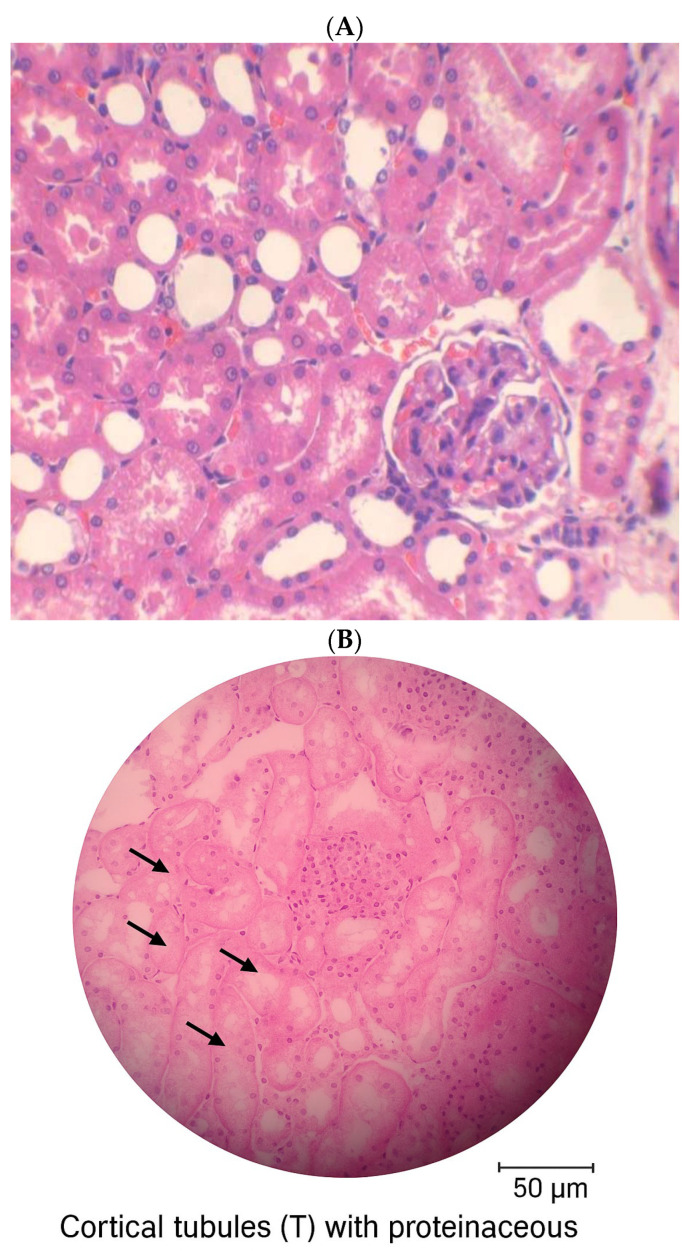
Histological analysis of kidneys perfused with TSP. No alterations observed in glomeruli or tubules in controls (**A**, Krebs–Henseleit). Kidneys perfused with TSP (1.0 μg/mL) showed intratubular and urinary space protein deposits (**B**, arrows). H&E staining, 400×.

Histopathological analysis revealed morphological alterations in kidneys perfused with TSP. Proteinaceous material was frequently observed within the tubules and urinary space, indicating tubular damage. Despite these changes, the medullary region remained preserved, showing normal Henle’s loops and collecting ducts (Figure 2).

**Figure 3:**
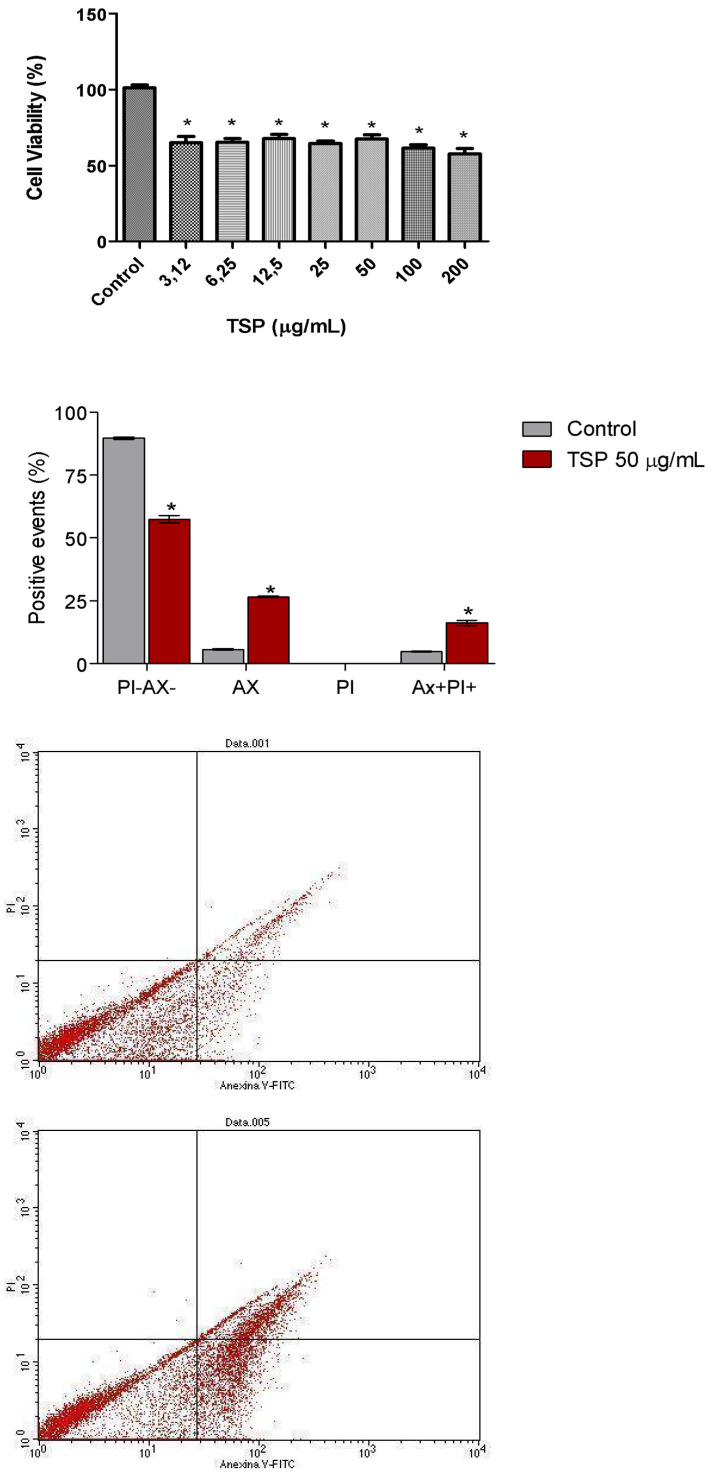
Cytotoxic and apoptotic effects of TSP from *Gracilaria cornea* on MDCK cells. Cell viability assessed by MTT assay after 24 h exposure to different concentrations of TSP. All concentrations reduced viability by ~50% compared with control. Bar colors represent treatment. Flow cytometry of MDCK cells treated with TSP (50 µg/mL) showing annexin V-FITC (apoptotic cells, red), propidium iodide (necrotic cells), and double-positive populations (apoptosis progressing to secondary necrosis). Data are mean ± S.E.M. of three independent experiments (*n* = 6). One-Way ANOVA, *p* < 0.05.

In vitro analysis using MDCK cells showed a concentration-dependent cytotoxic effect of TSP, with all concentrations (200 to 3.12 µg/mL) inducing approximately 50% cell mortality after 24 h of exposure (Figure 3).

Flow cytometric analysis of MDCK cells treated with TSP (50 μg/mL) indicated a predominantly apoptotic profile, as evidenced by annexin V-FITC staining. Propidium iodide staining remained low, suggesting intact membrane integrity. Some cells showed double labeling, indicating apoptotic cells progressing to secondary necrosis due to lack of phagocytic clearance.

## 3. Discussion

Although algae are largely consumed in food products worldwide, little is known about their kidney effects. Several studies have shown a range of biological activities of these marine organisms, but there is a shortage of studies using experimental models to evaluate their activities on renal function. To understand the effects of TSP on the renal system, an isolated kidney infusion system was used to evaluate the effects of these substances on renal function and morphology without any interference of systemic factors [17]. The chemical composition of TSP includes mainly 3,6-anhydro-α-L-galactose, with 6-O-methylgalactose, glucose, xylose and sulfate groups as secondary components [18].

The results obtained in this study demonstrated that the TSP causes distinct alterations on the kidney function, depending on the concentration. The administration of TSP at a concentration of 1 μg/mL in the renal perfusion system in rats showed no change in vascular parameters. On the other hand, at a concentration of 3 μg/mL, this treatment increased the perfusion pressure (Figure 1A) and renal vascular resistance (Figure 1B) at 90 and 120 min. However, the administration of TSP at 4.5 μg/mL, caused even more intense changes in these parameters, which were significantly increased as early as 60 min.

In contrast to the vascular parameters, the urinary flow (UF) and the glomerular filtration rate (GFR) underwent intense changes even at the lowest concentration of (1 μg/mL) of TSP. From the time of 90 min of infusion, the UF was significantly reduced (Figure 1C). At 90 and 120 min of perfusion, the UF was significantly increased by the administration of TSP at the concentration of 4.5 μg/mL, reaching values of 0.290 ± 0.017 and 0.331 ± 0.02 mL.g^−1^.min^−1^, respectively, corresponding to 52 and 73% of the internal control (C30 = 0.191 ± 0.03 mL.g^−1^.min^−1^). On the other hand, although at this concentration, the GFR was less affected, it decreased at 60 min (Figure 1D). The TSP caused renal tubular dysfunction by altering the tubular transport of electrolytes. The percentage of sodium transport (%TNa^+^) was decreased at all concentrations tested (Figure 1E). Nevertheless, no alterations were observed in the percentage of chloride (%TCl, Figure 1F) and potassium (%TK^+^, Figure 1G) transport on isolated kidney.

Histopathological analyses of the kidneys of TSP-perfused rats showed tubular alterations that suggest toxicity at all concentrations tested. Kidneys exposed to TSP exhibited marked accumulation of proteinaceous material within the urinary space and renal tubules. These histological findings may help explain the alterations observed in electrolyte transport during perfusion assays, likely reflecting tubular dysfunction induced by TSP exposure. In contrast, the medullary regions remained morphologically preserved, with no detectable changes in Henle’s loops or collecting ducts.

Tubular dysfunction can be caused by loss of tubular epithelial cell function [19]. In this study, TSP presented cytotoxicity against MDCK (distal tubular epithelial renal cell line), exhibiting a concentration-dependent decrease in the viability of cells treated for 24 h. MDCK cells constitute a cell line that have been extensively employed in the investigation of several cell processes, including epithelial transport and cell response to toxic agents [20]. Here, exposition to all concentrations of TSP (200, 100, 50, 25, 12.5, 6.25 e 3.12 µg/mL) caused mortality of 50% of MDCK cells (Figure 3), corroborating with the results obtained in vivo.

Flow cytometry was used to investigate the type of cell death caused by TSP. When MDCK cells were treated with TSP (50 μg/mL), they presented an apoptotic profile. Thus, a significant portion of the cells treated with TSP was labeled with annexin V-FITC as compared to the negative control (Figure 3). On the other hand, no significant percentage of cells were labeled with propidium iodide, indicating that the cells did not lose their membrane permeability.

Apoptosis is a form of programmed cell death characterized by phosphatidylserine externalization. In the absence of phagocytic clearance, apoptotic cells may progress to secondary necrosis. In this study, some TSP-treated cells exhibited double labeling with annexin V-FITC and propidium iodide, indicating incomplete apoptosis that likely advanced to necrosis in vitro. This pattern typically reflects apoptotic cells that, under physiological conditions, would be cleared by phagocytes. The absence of such cells in the in vitro environment likely accounts for the observed dual staining.

Recent studies have demonstrated that sulfated polysaccharides (SPs) extracted from marine algae can exert cytotoxic effects on renal cells. For instance, a study evaluated the impact of sulfated galactans from *Gracilaria fisheri* on human renal epithelial cells (HK-2). The results indicated that, at concentrations exceeding 1500 μg/mL, the SPs exhibited significant cytotoxicity, as evidenced by reduced cell viability. Furthermore, these SPs modulated the expression of apoptosis-related proteins, including caspase-3 and BCL-2, suggesting a mechanism involving programmed cell death [21].

Corroborating with the results found in this work, recent findings have emphasized the cytotoxic effects of algae in different cell lines. In fact, many species of algae of the genus *Gracilaria* have been recognized as potential sources of biologically active substances, including molecules with cytotoxic effects [22,23,24]. The antioxidant activity of polysaccharides derived from marine organisms has been extensively studied due to their therapeutic potential. A comprehensive review highlighted that polysaccharides extracted from various algae species exhibit a significant capacity to neutralize reactive oxygen species (ROS), protecting cells against oxidative stress. This property is particularly relevant in the context of kidney injury, where oxidative stress plays a crucial role in pathogenesis [25].

In a recent study, an extract obtained from *Gracilaria tenuistipitata* presented cytotoxic effects against oral cancer cells incubated for 24 h with the extract and evaluated by MTT. The authors demonstrated that the cytotoxic effect was concentration-dependent and involved mechanisms linked to the molecular pathway of caspases [22]. In a previous study, the cytotoxic effect of a brown algae extract (*Sargassum muticum*) on breast cancer cell lines and renal monkey cells was evaluated [9]. They demonstrated that the extract presented cytotoxic effects against the tumor, but not against normal renal cells. In terms of mechanism, a study with fucoidan, demonstrated its cytotoxic effect on gastric adenocarcinoma cell cultures by inducing apoptosis, which was mediated by a decrease in the expression of antiapoptotic genes, such as Bcl-2 and Bcl-xL, in addition to activation of caspases [26].

The cytotoxic effects observed in MDCK cells upon treatment with total sulfated polysaccharides (TSPs) from *Gracilaria cornea* suggest the activation of intrinsic apoptotic pathways. Studies have shown that sulfated polysaccharides from marine algae can modulate the expression of Bcl-2 family proteins, leading to mitochondrial membrane permeabilization and subsequent activation of caspases. For instance, a methyl alcohol extract of the green alga *Ulva linza* (formerly *Enteromorpha linza*) was found to upregulate the pro-apoptotic protein Bax while downregulating the anti-apoptotic protein Bcl-2, resulting in cytochrome c release and activation of caspase-9 and caspase-3 in Hep3B cells. Similarly, fucoidan, a sulfated polysaccharide from brown algae, has been reported to induce apoptosis in human leukemia cells through the activation of caspases and degradation of PARP. These findings align with our observations, indicating that TSP may trigger apoptosis via the mitochondrial pathway, involving modulation of Bcl-2 family proteins and activation of caspases [22,26,27,28].

Finally, studies have demonstrated the cytotoxic effects of seaweed polysaccharides on tumor cell lines. A sulphated polysaccharide extracted from *Palisada perforata* (formerly *Laurencia papillosa*) (Rhodophyta) induced apoptosis in a lineage of breast cancer cells [8]. In a previous study, the apoptotic effect of a polysaccharide derived from a seaweed extract was evaluated in a gastric carcinoma cell culture. They demonstrated that this effect was associated with the generation of reactive oxygen species and activation of caspases, resulting in the suppression of the proliferation of cancerous cells. Then, in a study carried out with a polysaccharide obtained from *Sargassum*, they demonstrated that this substance has an antiproliferative effect against cancer cell lines that is associated with caspase-mediated apoptosis [29,30].

In addition to their antioxidant effects, marine polysaccharides have shown therapeutic potential in various chronic diseases. A recent review discussed the pharmacological applications of SPs derived from marine algae, highlighting their anti-inflammatory, antiviral, and anticancer activities. These properties are attributed to the ability of SPs to modulate cellular signaling pathways, influencing processes such as apoptosis and cell proliferation [27,31,32].

Together, the results obtained in this work suggest that the effects of TSP on the perfusion system lead to acute renal failure (ARF) as exposure to this substance altered electrolyte transport and decreased the renal function. Suggestively, this renal failure could be caused by induction of apoptosis of renal epithelial cells promoted by the polysaccharides. 

Polysaccharides extracted from marine algae have been explored for various biomedical applications, including controlled drug delivery systems and tissue engineering. Their bioactive properties, such as biocompatibility and biodegradability, make them promising candidates for the development of therapeutic biomaterials. These characteristics are particularly relevant in renal therapies, where targeted delivery of therapeutic agents can improve clinical outcomes [31,33].

Understanding the pharmacokinetic properties of sulfated polysaccharides (TSPs) is crucial for evaluating their therapeutic potential. Although this study focused on the renal effects of TSP, further investigation is necessary to determine its absorption, distribution, metabolism, and excretion (ADME) characteristics. Marine-derived polysaccharides, such as fucoidans, are known to exhibit low oral bioavailability due to their high molecular weight and strong negative charge, which limits gastrointestinal absorption. Strategies such as chemical modification, nanoparticle encapsulation, and alternative administration routes have been explored to enhance their bioavailability. Future research should focus on the pharmacokinetic characterization of TSP to optimize its therapeutic efficacy [34,35]. Interactions between TSP and renal cells, including the possibility of biofilm formation or the development of physical barriers that may influence the interpretation of functional outcomes. This hypothesis is supported by previous reports involving high molecular weight polysaccharides.

Our results indicate that TSP increases renal vascular resistance with a concomitant decrease in GFR, while the highest concentration elevates urinary flow. A plausible explanation is that TSP interferes with endothelial–tubular interactions. Reduced NO availability and/or endothelin-mediated vasoconstriction could account for the hemodynamic component (↑RVR, ↓GFR). In parallel, epithelial injury—supported by annexin V/PI staining and intratubular protein deposits—likely compromises sodium and water reabsorption, resulting in higher UF and altered electrolyte transport. This overall pattern is compatible with a biphasic dose–response, where vasoconstriction predominates at low concentrations and tubular dysfunction becomes prominent at higher doses.

These findings are derived from ex vivo and in vitro models and should therefore be considered hypothesis-generating. Human relevance will depend on factors such as exposure route, bioavailability, molecular size, and the degree/pattern of sulfation of TSP. Because pharmacokinetics, biodistribution, and immune responses remain unknown, no conclusions about clinical safety or therapeutic applicability can be drawn at this stage. Instead, our results highlight potential renal liabilities that warrant further in vivo confirmation.

Methodologically, MTT primarily reflects mitochondrial activity, and although annexin V/PI provided complementary information, additional assays—such as LDH release, caspase-3/9 activity, Bcl-2/Bax expression, ROS generation, and mitochondrial membrane potential—are needed to fully characterize apoptosis versus necrosis. On the renal side, quantification of Na^+^/K^+^-ATPase, NKCC2, NCC, ENaC, AQP1/AQP2, and eNOS, as well as vasoreactivity testing of renal arteries, would further clarify the mechanistic link between hemodynamics and tubular transport. In vivo studies are also essential to evaluate systemic kidney function (creatinine, BUN, fractional excretion), hemodynamics, and cortical/medullary histology. Finally, expanded chemical characterization of TSP (endotoxin content, sulfate distribution, molecular weight profile) will be critical to strengthen reproducibility and translational relevance.

When comparing our results with existing studies, it becomes evident that sulfated polysaccharides from different *Gracilaria* species exhibit both overlapping and distinct biological effects. For instance, sulfated galactans from *G. birdiae* have been reported to modulate hemostasis and exert anticoagulant activity without clear nephrotoxicity [16]. In contrast, extracts from *G. tenuistipitata* demonstrated cytotoxic and pro-apoptotic effects in oral cancer cell lines, mediated by caspase activation [22]. Similarly, sulfated polysaccharides from *G. fisheri* induced apoptosis in renal epithelial cells (HK-2) at high concentrations, reinforcing their potential for renal toxicity [21]. These findings support the notion that although sulfated polysaccharides share structural similarities, their biological impact may vary depending on algal species, sulfation pattern, and molecular weight. Within this context, our study provides the first systematic evidence that TSP from *G. cornea* can alter renal hemodynamics and tubular function, while also inducing apoptosis in renal epithelial cells.

In conclusion, TSP from *Gracilaria cornea* induced significant alterations in renal physiology and morphology, as well as notable cytotoxic effects on renal epithelial cells, suggestive of apoptosis-mediated cell death. These findings align with recent studies highlighting the capacity of sulfated polysaccharides from marine algae to modulate cellular pathways involved in oxidative stress, inflammation, and apoptosis. However, further investigations are necessary to fully elucidate the specific signaling pathways underlying the renal effects of TSP, as well as to assess their potential as therapeutic agents in nephrology and related fields. The incorporation of advanced analytical methods and standardized bioactivity assays will be essential to establish the safety, efficacy, and therapeutic relevance of these natural compounds.

## 4. Conclusions

TSP from *Gracilaria cornea* induced alterations in renal hemodynamics and tubular transport in isolated perfused kidneys and demonstrated cytotoxic effects on renal epithelial cells in vitro, consistent with apoptosis-mediated injury. These findings provide preliminary evidence of potential renal toxicity of marine-derived sulfated polysaccharides. However, they should be interpreted as an initial step, underscoring the need for further mechanistic assays and in vivo studies to establish safety, efficacy, and possible biomedical applications.

## 5. Materials and Methods

### 5.1. Study Compliance

All procedures were conducted in accordance with the guidelines of Toxins for research involving natural products and systems pharmacology. Animal experiments were approved by the Ethics Committee for Animal Research of the Federal University of Ceará (CEUA/UFC) under protocol number 79/08, date 6 August 2013.

### 5.2. Algal Material and Extraction of Sulfated Polysaccharides

Specimens of *Gracilaria cornea* were collected from Flecheiras Beach (Ceará, Brazil). Samples were transported to the Carbohydrates and Lectins Laboratory (CarboLec) at the Department of Biochemistry and Molecular Biology, Federal University of Ceará. After manual removal of epiphytes and thorough washing with distilled water, the algae were stored at −20 °C. A voucher specimen was deposited in the Herbarium Prisco Bezerra under accession number 34739.

Total sulfated polysaccharides (TSPs) were extracted from 5 g of dried algal tissue using enzymatic digestion with papain (6 h, 60 °C) in 100 mM sodium acetate buffer (pH 5.0) containing cysteine and EDTA (5 mM), following established protocols [36]. Carbohydrate content was quantified using the phenol–sulfuric acid method, and sulfate content was assessed after hydrolysis (1 M HCl, 110 °C, 5 h) using the gelatin–barium method [37]. Protein contamination was evaluated by Coomassie Brilliant Blue G-250 dye binding. The absence of nucleic acid contamination was verified using a NanoDrop 2000 spectrophotometer (Thermo Fisher Scientific, Waltham, MA, USA), and sample purity was assessed by 0.5% agarose gel electrophoresis [36,37].

### 5.3. Isolated Kidney Perfusion

Male Wistar rats (260–320 g) were fasted for 24 h with free access to water prior to the procedure. Anesthesia was induced via intraperitoneal administration of sodium pentobarbital (50 mg/kg). The right kidney was surgically isolated, and the renal artery was cannulated through the mesenteric artery, preserving blood flow [17].

Kidneys were perfused using modified Krebs–Henseleit solution (MKHS) containing 114 mM NaCl, 4.96 mM KCl, 1.24 mM KH_2_PO_4_, 0.5 mM MgSO_4_·7H_2_O, 2.10 mM CaCl_2_, and 24.99 mM NaHCO_3_. Additives included 6% bovine serum albumin (BSA, fraction V), 0.075 g urea, 0.075 g inulin, and 0.15 g glucose per 100 mL. The pH was adjusted to 7.4. In each experiment, 100 mL of MKHS was recirculated for 120 min [38].

The perfusion pressure (PP) was measured at the tip of the stainless-steel cannula in the renal artery. Urine and perfusate samples were collected at 10 min-intervals for analysis of sodium, potassium and chloride levels by ion-selective electrodes (RapidChem 744, Bayer Diagnostics, Newbury, UK); inulin [17]; and osmolality, which was measured in a vapor pressure osmometer (Wescor 5100C, Logan, UT, USA). Solubilization process of the SP in sterile isotonic saline solution (0.9% NaCl), including pH adjustment and precautions taken to ensure full homogenization prior to renal perfusion. The viscosity of the solution was also monitored to ensure consistency across experiments.

The TSP (1 μg/mL, 3 μg/mL e 4.5 μg/mL) was added to the system 30 min after the start of each renal perfusion. The following parameters were measured: perfusion pressure (PP), renal vascular resistance (RVR), urinary flow (UF), glomerular filtration rate (GFR), and tubular transport percentages of sodium (%TNa^+^), potassium (%TK^+^), and chloride (%TCl^−^) [38]. Values were compared to the internal control recorded at 30 min *(n* = 6). Both kidneys (right and left) were fixed in formaldehyde for histological processing, which included dehydration, clearing, and sectioning into 3 μm-thick slices.

Following perfusion, kidneys were fixed in 10% buffered formalin, dehydrated, cleared, and embedded in paraffin. Sections (3 µm thick) were stained with hematoxylin and eosin (H&E) and examined under light microscopy for histopathological changes.

### 5.4. In Vitro Cytotoxicity Assays

Madin-Darby Canine Kidney (MDCK) epithelial cells were maintained in RPMI-1640 medium (Sigma-Aldrich, St. Louis, MO, USA) supplemented with 10% fetal bovine serum and 1% penicillin–streptomycin, at 37 °C in a 5% CO_2_ humidified incubator. Cells were seeded in 96-well plates at a density of 1 × 10^5^ cells/mL and allowed to adhere for 24 h before treatment.

For the MTT assay, cells were treated with total sulfated polysaccharides (TSPs) extracted from Gracilaria cornea at concentrations of 200, 100, 50, 25, 12.5, 6.25, and 3.12 µg/mL for 24, 48, and 72 h. Following treatment, plates were centrifuged at 1500 rpm for 15 min, and 100 µL of culture supernatant was discarded. Next, 500 µg/mL of MTT solution (Sigma-Aldrich, USA) dissolved in PBS was added to each well, and the plates were incubated for 4 h at 37 °C. After incubation, plates were centrifuged at 3000 rpm for 10 min, the supernatant was removed, and 10% sodium dodecyl sulfate (SDS) in 0.01 N HCl was added to solubilize the formazan crystals. The plates were kept in a 37 °C incubator with 5% CO_2_ for an additional 17 h. Absorbance was measured at 570 nm using a Biochrom^®^ Asys Expert Plus microplate reader (Biochrom Ltd., Cambridge, UK). Cell viability was calculated as a percentage relative to the mean absorbance of the untreated control group (considered as 100% viability) [39]. Data were expressed as mean ± standard error of the mean (SEM) from three independent experiments. Non-linear regression analysis for IC_50_ determination was performed using GraphPad Prism software (version 9.5.1). The passage number of MDCK cells used for the assays ranged from 4 to 6. Control treatments included untreated cells as negative controls.

### 5.5. Flow Cytometry Analysis of Cell Death

To characterize the type of cell death induced by TSP, MDCK cells were treated with 50 μg/mL of TSP for 12 h, then labeled with LDH release V-FITC and propidium iodide (PI) according to manufacturer’s instructions (BD Pharmingen, San Diego, CA, USA). Cells were analyzed using a FACS Calibur flow cytometer (Becton-Dickinson, San Jose, CA, USA) and data were processed with CellQuest software, version 5.2. Cell populations were classified as viable (Annexin V^−^/PI^−^), early apoptotic (Annexin V^+^/PI^−^), or necrotic/late apoptotic (Annexin V^+^/PI^+^).

### 5.6. Statistical Analysis

Data are expressed as mean ± standard error of the mean (SEM). In vivo experiments were performed in six animals per group, while in vitro assays were conducted in triplicate. Statistical differences were determined using one-way analysis of variance (ANOVA), with significance accepted at *p* < 0.05.

## Data Availability

The data presented in this study are openly available in [Repositório UFC] at [https://repositorio.ufc.br/handle/riufc/11457], reference number [11457], accessed on 30 September 2025.
